# The feeding habit of sea turtles influences their reaction to artificial marine debris

**DOI:** 10.1038/srep28015

**Published:** 2016-06-16

**Authors:** Takuya Fukuoka, Misaki Yamane, Chihiro Kinoshita, Tomoko Narazaki, Greg J. Marshall, Kyler J. Abernathy, Nobuyuki Miyazaki, Katsufumi Sato

**Affiliations:** 1 Atmosphere and Ocean Research Institute, The University of Tokyo, Kashiwa, Chiba, 277-8564, Japan; 2 National Geographic Remote Imaging, 1145 17th Street NW Washington, D.C., 20036, United States of America; 3 Japan Marine Science Foundation, 1-1-1 Ikenohata, Taito-ku, Tokyo, 110-0008, Japan

## Abstract

Ingestion of artificial debris is considered as a significant stress for wildlife including sea turtles. To investigate how turtles react to artificial debris under natural conditions, we deployed animal-borne video cameras on loggerhead and green turtles in addition to feces and gut contents analyses from 2007 to 2015. Frequency of occurrences of artificial debris in feces and gut contents collected from loggerhead turtles were 35.7% (10/28) and 84.6% (11/13), respectively. Artificial debris appeared in all green turtles in feces (25/25) and gut contents (10/10), and green turtles ingested more debris (feces; 15.8 ± 33.4 g, gut; 39.8 ± 51.2 g) than loggerhead turtles (feces; 1.6 ± 3.7 g, gut; 9.7 ± 15.0 g). In the video records (60 and 52.5 hours from 10 loggerhead and 6 green turtles, respectively), turtles encountered 46 artificial debris and ingested 23 of them. The encounter-ingestion ratio of artificial debris in green turtles (61.8%) was significantly higher than that in loggerhead turtles (16.7%). Loggerhead turtles frequently fed on gelatinous prey (78/84), however, green turtles mainly fed marine algae (156/210), and partly consumed gelatinous prey (10/210). Turtles seemed to confuse solo drifting debris with their diet, and omnivorous green turtles were more attracted by artificial debris.

Artificial marine debris is now recognized worldwide as a significant stressor for marine wildlife^1^. Ingestion of artificial debris is one of the threats of marine pollution along with entanglement, bioaccumulation and changes to the integrity and functioning of habitats^2^. Artificial debris ingestion can have lethal and sub-lethal effects such as gut obstruction^3^,^4^, reduced food intake^5^ and transfer of toxic compounds^6^,^7^,^8^ on marine wildlife^9^. Laist^10^ noted that 177 marine species ingested artificial debris including mammals^11^, seabirds^12^,^13^,^14^, fishes^15^,^16^,^17^ and sea turtles^18^,^19^.

Six of 7 species of sea turtles have been found to ingest artificial debris as early as the 1980s, with exception of the flatback turtles^18^. Since then, ingestion of artificial debris by sea turtles has been investigated on local or regional scales by gut contents analyses^3^,^20^,^21^. The percentage of the turtles ingesting artificial debris varied among the study areas^19^. Schuyler *et al*.^19^ suggested that this inter-regional variation might be caused by the difference of feeding habits in each region. Therefore, it is important to study debris ingestion as a component of diet in a variety of areas.

Previous studies about the debris ingestion have been focused on examining the characteristics of artificial debris ingested by sea turtles^18^,^22^. Thus, the trends of ingested debris such as material types (soft plastic^19^) and colors (white and transparent^23^) have been well documented. On the other hand, reaction to artificial debris and the process of the debris ingestion remained unknown because it is difficult to observe under natural conditions. There are two possible processes that could lead to ingesting artificial debris: one is ingestion by confusing debris with typical diet items of sea turtles, and the other is accidental ingestion when they consumed natural diet items. In addition, Schuyler *et al*.^19^ reported that the herbivore species (green turtle) may be more likely to ingest debris than carnivorous species (loggerhead turtle). This could be because the herbivore species is more likely to eat artificial debris due to its similarity to their diets. However, there is no evidence whether herbivore species are more attracted (high encounter-ingestion ratio) by debris relative to carnivorous species. Hence, investigating responses of turtles when they encounter artificial debris under natural conditions is important for understanding debris ingestion by sea turtles.

Animal-borne video cameras have been developed in recent decades^24^. The video cameras have been deployed on a number of taxa, including sea turtles^25^, cetaceans^26^, pinnipeds^27^, seabirds^28^,^29^ and fish^30^, and they provided novel insights into foraging behavior^25^,^26^,^27^,^30^, foraging habitat^28^,^30^ and prey density^31^. Previous studies using animal-borne video cameras have focused on foraging ecology, however, it is also suitable for studying how animals react to artificial debris encountered under natural conditions. For example, Narazaki *et al*.^32^ indicated one example in which a loggerhead turtle approached to a plastic bag drifting in mid-water although this turtle did not bite it. It indicates that loggerhead turtles have ability to distinguish the debris from their diet. Therefore, using animal-borne video cameras, we can investigate the responses of turtles when they encounter artificial debris and compare to encounter-ingestion ratios of artificial debris between species.

The Sanriku Coast in the Japanese archipelago, located in a temperate area of the northwest Pacific Ocean, is a seasonal foraging area for loggerhead turtles *Caretta caretta*^33^ and green turtles *Chelonia mydas*^34^. Previous studies involving gut content analysis noted that loggerhead and green turtles in Japan are considered benthic-carnivore and herbivore, respectively^35^,^36^. However, stable isotope analysis suggested that some loggerhead and green turtles nesting in Japanese rookeries, where the turtles migrated to the Sanriku Coast was born, display omnivory^37^,^38^. On the Sanriku Coast, although there is no detailed information about diet composition, Narazaki *et al*.^32^ reported that loggerhead turtles mainly foraged on gelatinous prey in mid-water. It is inferred that the Sanriku Coast is an abundant supply of gelatinous prey because some currents occupy this area alternatively and interact with each other^39^. Hence it is possible that the turtles migrating to the Sanriku Coast display more planktivory compared to other areas. Anecdotally, it is considered that sea turtles confuse marine debris with jellyfish. Thus, the turtles may ingest a significant amount of artificial debris in this area. This situation offered the opportunity to examine the difference in behavioral response between these species to encountering artificial debris.

In this study, we conducted feces and gut contents analyses to understand the regional diet of loggerhead and green turtles migrating to the Sanriku Coast. Then we described the ingested artificial debris in feces and gut contents. Animal-borne video cameras were used to examine their responses when they encountered artificial debris. Finally, we evaluate the difference of encounter-ingestion ratios of the debris between loggerhead and green turtles in relation to their feeding habit of each species.

## Results

### Feeding habit and observed artificial debris in feces and gut contents

#### Feces contents

During the study period from 2012 to 2015, we collected feces samples from 28 loggerhead and 25 green turtles. SCLs of turtles, from which feces samples were collected, were 74.9 ± 6.8 cm (range = 62.3–89.0 cm) for loggerhead and 49.1 ± 12.9 cm (range = 38.0–90.9 cm) for green turtles. Mean masses of feces sample from loggerhead and green turtles were 112.4 ± 147.1 g (range = 0–623 g, n = 28) and 40.9 ± 57.5 g (range = 1.9–210.6 g, n = 25), respectively. Malacostraca, Phaeophyceae, natural debris (others) and artificial debris were the most frequent types of materials for loggerhead turtles, found in more than 35% of the fecal samples (equation (1)). Monocotyledoneae, Phaeophyceae, Maxillopoda, natural debris (bird feather) and artificial debris were found in more than half of green turtles (equation (1), Table 1). In %mass (equation (2)), Echinoidea, Maxillopoda and Gastropoda were dominant for loggerhead turtles, and Phaeophyceae and artificial debris were dominant for green turtles (Table 1).

#### Gut contents

Samples of gut contents were obtained from 13 loggerhead and 10 green turtles from 2012 to 2015. SCLs of turtles, from which gut samples were collected, were 73.7 ± 5.6 cm (range = 65.2–82.3 cm) for loggerhead and 44.2 ± 2.3 cm (range = 40.5–48.4 cm) for green turtles. Mean masses of gut contents collected from loggerhead and green turtles were 266.8 ± 197.1 g (range = 16.7–650.6 g, n = 13) and 278.9 ± 180.3 g (range = 24.7–517.2 g, n = 10), respectively. Gastropoda, Maxillopoda, natural debris (wood/leaves) and artificial debris were found in more than half of loggerhead turtles (equation (1)), and Plantae, Maxillopoda and artificial debris were found in most of green turtles (>80%, equation (1), Table 1). In %mass (equation (2)), Echinoidea and Maxillopoda were dominant for loggerhead turtles, and Phycophyta was dominant for green turtles (Table 1). In this study, no turtles exhibited gut obstruction due to the ingested artificial debris.

#### Observed artificial debris

Frequency of occurrences of artificial debris (%F: equation (1)) in feces and gut contents collected from loggerhead turtles were 35.7% (10/28) and 84.6% (11/13), respectively (Table 1). Artificial debris was recovered from all green turtles in both feces (25/25) and gut contents (10/10) (equation (1), Table 1). The turtles migrating to Sanriku Coast displayed high %F of artificial debris in gut contents (loggerhead turtles: 84.6%, n = 13; green turtles: 100%, n = 10) relative to previous studies of both species around world (loggerhead turtles: 27.2%, n = 923; green turtles: 41.5%, n = 754)^19^,^23^,^40^,^41^,^42^,^43^ and in Japan (loggerhead turtles: 25.9%, n = 162; green turtles: 36.6%, n = 235)^36^,^44^,^45^.

In types of debris, soft plastic debris was most frequently found in both of feces and gut contents (Table 2), and there are significant differences between types of debris (Kruskal-Wallis rank sum test; χ^2^ = 79.98, p < 0.001 for feces, χ^2^ = 35.31, p < 0.001 for gut contents). In term of debris color, transparent debris was most observed in both feces and gut contents (Table 2). A significant difference was observed between colors of debris in gut contents (Kruskal-Wallis rank sum test: χ^2^ = 11.10, p = 0.01), whereas no significant difference was found in feces (Kruskal-Wallis rank sum test: χ^2^ = 7.39, p = 0.06).

Mass percentages of artificial debris in feces and gut contents (equation (2)) were 1.5% (range = 0–100%, n = 28) and 3.6% (range = 0–37.8%, n = 13) for loggerhead turtles, respectively, and 38.6% (range = 6.0–100%, n = 25) and 14.3% (range = 0.2–59.9%, n = 10) for green turtles, respectively (Table 1). Wet masses of artificial debris of green turtles (feces; 15.8 ± 33.4 g, gut; 39.8 ± 51.2 g) were significantly heavier than that of loggerhead turtles (feces; 1.6 ± 3.7 g, gut; 9.7 ± 15.0 g, Wilcoxon rank sum test; W = 611.5, p < 0.001 for feces, W = 105.5, p = 0.01 for gut contents).

### Feeding habits revealed by animal-borne video camera

Animal-borne video cameras were attached on the carapace of 15 loggerhead and 10 green turtles, however, in the case of 5 loggerhead turtles and 4 green turtles, we did not obtain the video data because of problems before video recording started (the data loggers displaced/detached from 7 turtles and 2 turtles were recaptured). As a result, a total of 60 and 52.5 hours of video data were obtained from 10 loggerhead and 6 green turtles, respectively (Table 3). SCLs of the instrumented turtles were 78.2 ± 5.1 cm (range = 70.0–85.0 cm, n = 10) for loggerhead turtles and 55.2 ± 13.8 cm (range = 44.5–81.0 cm, n = 6) for green turtles.

**Figure 1:**
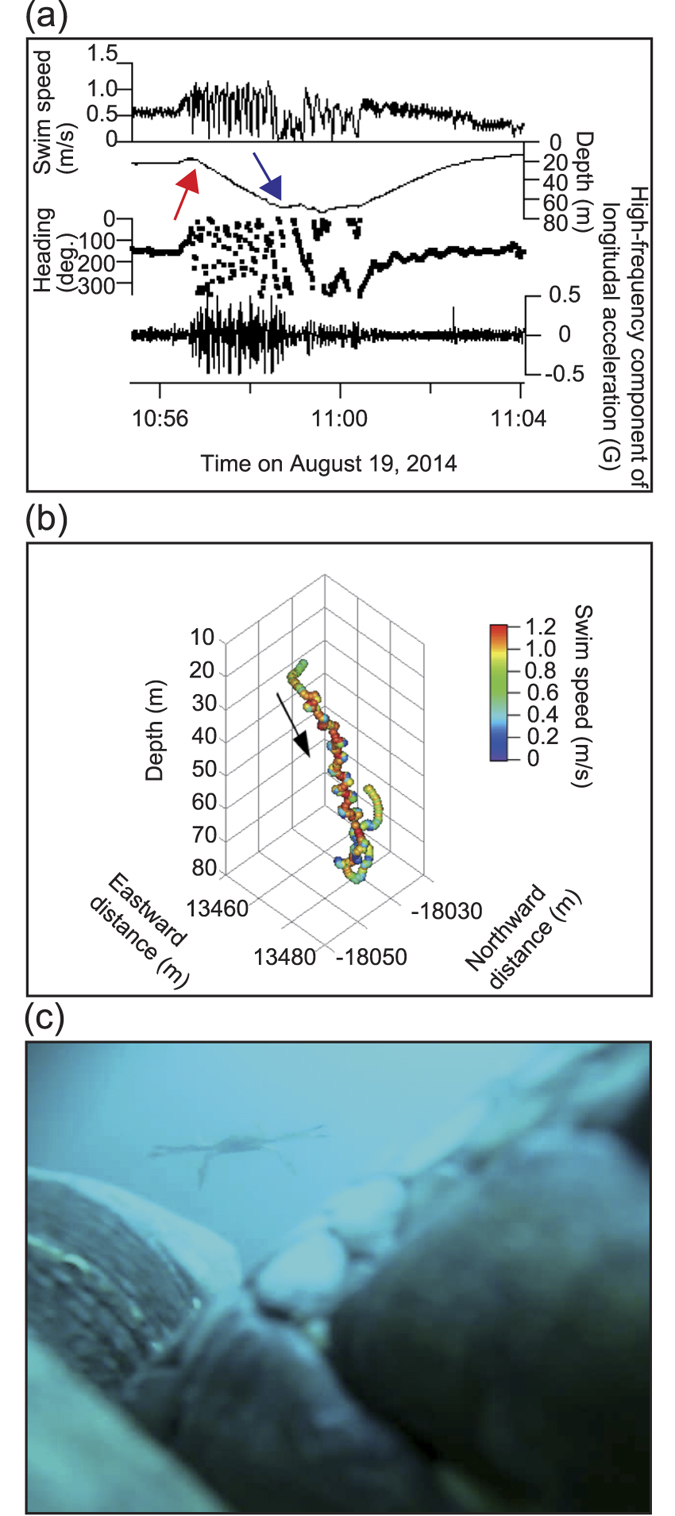
A loggerhead turtle (L1410) pursuing a blue crab. (**a**) Time-series data and (**b**) 3D path movement during a loggerhead turtle chasing a blue crab. Red and blue arrows indicate the time when the turtle encountered and captured the crab, respectively. Black arrow shows the direction of the movement. (**c**) A picture of a blue crab eaten by the loggerhead turtle.

**Figure 2:**
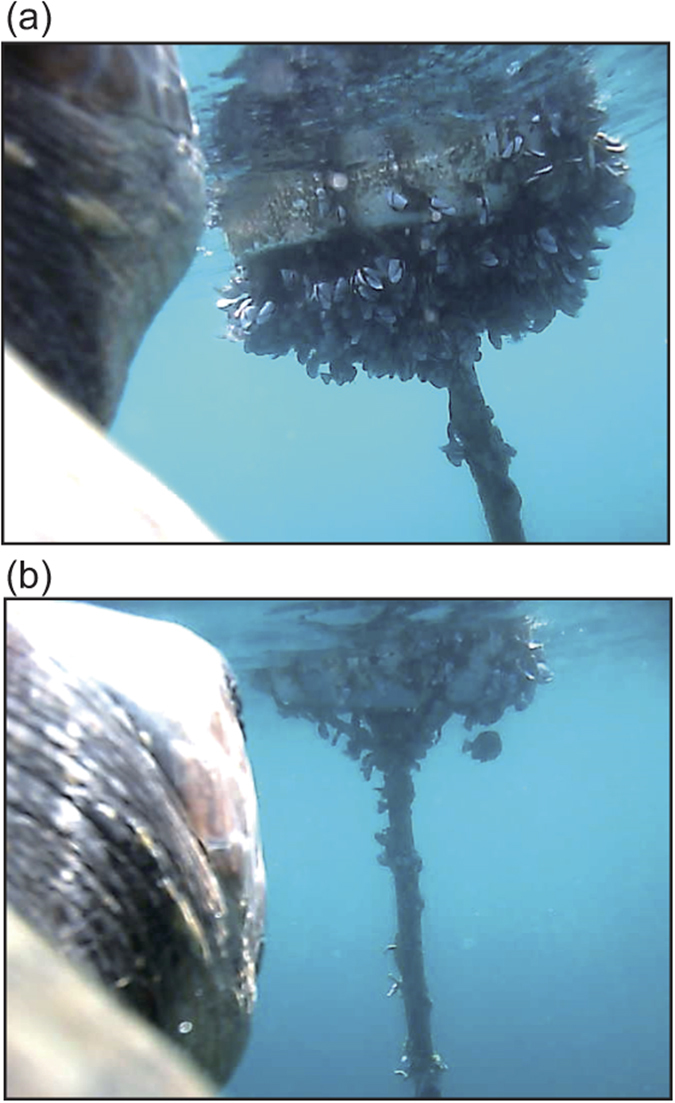
A loggerhead turtle (L1410) feeds on gooseneck barnacles on a Styrofoam buoy. (**a**) A picture when the turtle encounter the buoy at 09:05 on August 18, 2014. (**b**) Twenty-two minutes later, the turtle consumed almost all of the barnacles.

In the loggerhead turtles, a total of 84 feeding events were recorded from 6 turtles (Table 3). Mean feeding depth was 18.3 ± 8.5 m (n = 84). Seventy-eight out of the 84 events were associated with gelatinous prey, such as Hydrozoa (67 events) and Scyphozoa (11 events) (Table 4). One loggerhead turtle (L1410) chased a blue crab (*Portunus sp*.) from 20 m to 70 m deep and fed (Fig. 1, Movie S1). Mean swim speed of this turtle during the chasing period was 0.88 ± 0.27 m s^−1^ (Maximum 1.26 m s^−1^) while mean cruise speed was 0.49 m s^−1^. The same individual fed on gooseneck barnacles on a Styrofoam buoy over a period of 20 minutes (Fig. 2, Movie S2). Loggerhead turtles also fed on seaweed (1 time), natural debris (leaf; 1 time) and artificial debris (2 times).

For green turtles, a total of 210 feeding events were recorded from all 6 turtles (Table 3). Mean feeding depth was 5.4 ± 4.8 m (n = 210), and it is significantly shallower than feeding depth of loggerhead turtles (Wilcoxon rank sum test: W = 2613.5, p < 0.001). 156 out of the 210 events were marine algae (Table 4), such as Florideophyceae (73 events), Phaeophyceae (29 events), Ulvophyceae (8 events) and unknown (46 events). In addition, three green turtles (G0718, G1454, G1514) fed on jellyfish (Scyphozoa: 6 events, Movie S3), and one green turtle (G1514) fed on salps near the surface (*Thetys vagina*: 2 events, Movie S4). Green turtles also ingested natural debris such as bird feather and wood (25 times) and artificial debris (21 times, Movie S5).

### Process of artificial debris ingestion under natural condition

**Figure 3:**
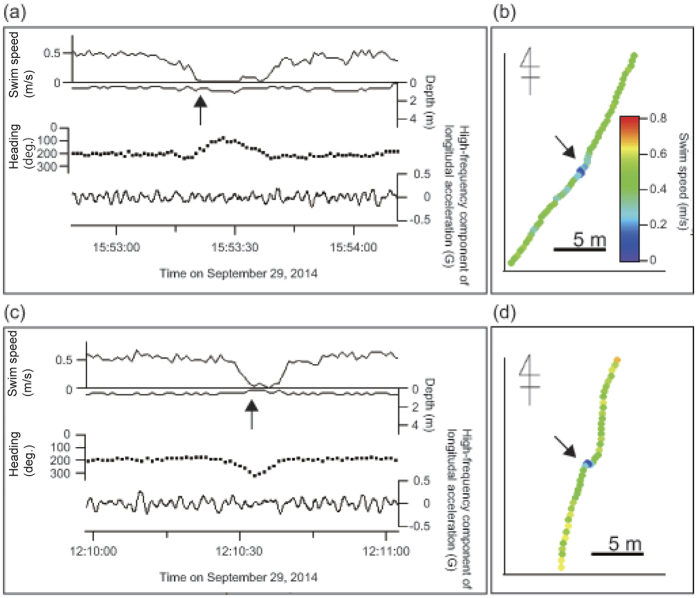
Time-series data and horizontal movements during feeding events. A green turtle (G1454) fed on a jellyfish (**a,b**) and ingested a plastic bag (**c,d**). Black arrows indicate the moment of capture/ingest.

A total of 46 artificial debris were encountered by 7 loggerhead and 4 green turtles, and 23 out of the 46 artificial debris were ingested (Table 5). Twenty-two out of 23 ingestion events were deliberate ingestion of isolated drifting debris. In the remaining case, a loggerhead turtle (L1410) ingested Styrofoam incidental to feeding on gooseneck barnacles (Fig. 2, Movie S2). Mean encounter depth of artificial debris were 14.0 ± 19.5 m for loggerhead turtles (n = 12) and 1.6 ± 2.3 m for green turtles (n = 34). Mean ingestion depth of debris were 2.9 ± 0.8 m for loggerhead turtles (n = 2) and 1.5 ± 1.6 m for green turtles (n = 21). Soft plastic debris (17/23) and transparent debris (15/23) were most ingested (Table 5). When the loggerhead and green turtles approached the debris, the movement pattern resembled that when they foraged on gelatinous prey (Fig. 3, change of travelling direction and reduced swim speed as described by Narazaki *et al*.^32^). Hourly encounter ratio of artificial debris in both loggerhead and green turtles were 0.2 times and 0.65 times, respectively. The GLM revealed that encounter ratio of artificial debris was related to species (AIC = 89.17, Table S1). The encounter-ingestion ratios of loggerhead and green turtles were 16.7% (2/12) and 61.8% (21/34), respectively, and this ratio was significantly different between species (Fisher’s exact test: p = 0.02).

## Discussion

The feces and gut contents analyses indicated that loggerhead and green turtles at the Sanriku Coast were primarily benthic carnivore and herbivore, respectively, which coincided with previous studies in Japan^35^,^36^. However, the loggerhead turtles with animal-borne video camera did not forage on benthic animals during the recorded periods, but instead foraged primarily on gelatinous prey (78 times in 60 hours) in mid-water. Although the green turtles mainly foraged on marine algae (156 times in 52.5 hours), they also foraged on gelatinous prey (8 times in 52.5 hours). These feeding habits in two species (loggerhead turtles: planktivore, green turtles: omnivore) were similar throughout the study period from 2007 to 2015. Previous studies have shown evidence of gelatinous prey feeding in loggerhead and green turtles using animal-borne video camera^25^,^32^,^46^. Recent stable isotope analyses indicated that the gelatinous prey was more important diet for loggerhead and green turtles than that previously thought^37^,^38^,^47^,^48^. Therefore, our results and a previous study^32^ indicated that feces and gut contents analyses underestimated the importance of gelatinous prey in the diet. The Sanriku Coast is known as one of the highest productivity marine areas in Japan because the Tsugaru Warm Current flowing southward, the cold nutrient-rich Oyashio water, and the warm Kuroshio water occupy this area alternatively and interact with each other^39^. It is inferred that there is an abundant supply of gelatinous prey, and hence, the turtles could forage on large numbers of these organisms. Additionally, it is noted that the loggerhead turtle which foraged on a blue crab in midwater invested a relatively large amount of effort to capture this one prey item. This suggests that blue crab is an attractive diet item (e.g. nutrient-rich relative to gelatinous prey) for loggerhead turtles, worth the extra foraging cost.

This study revealed that the transparent soft plastic was the major debris ingested by sea turtles along the Sanriku Coast. This trend is similar to previous results of gut contents analyses^19^,^23^. In addition, video data in this study indicated that the turtles usually ingested drifting debris deliberately, and the movement pattern was similar to approaches on gelatinous prey by loggerhead turtles (change of travelling direction and decelerating swim speed as described by Narazaki *et al*.^32^). There is evidence that sea turtles primarily use visual cues to locate prey^32^. Our results indicate that the turtles confused artificial debris with gelatinous prey visually. Additionally, we found that both species of turtles migrating to the Sanriku Coast had higher %F of debris ingestion than other study areas. This high ingestion rate may be due to a higher ratio feeding on gelatinous prey. Video data suggested that loggerhead turtles foraged frequently on gelatinous prey at the Sanriku Coast. Although green turtles mainly consumed marine algae in this study, stable isotope analysis suggests that green turtles in this area also fed on jellyfish considerably (Fukuoka *et al*. unpublished data). The transparent drifting soft plastic debris which is the most ingested by turtles has similar characteristics to gelatinous prey (e.g. colors, drifting in mid-water), and thus, the turtles might ingest lots of debris in this area. Additionally, it was reported that loggerhead turtles in this area foraged on gelatinous prey during the travelling period^32^. In this study, green turtles foraged on gelatinous prey when they were swimming near the surface (Fig. 3a). Furthermore, both loggerhead and green turtles released in this area travelled more than hundreds to thousands of kilometers seasonally^33^,^34^. Especially for green turtles, these movements are much longer than seen in other areas^34^. If the turtles are particularly prone to mistake debris for gelatinous prey during the travelling period, these migration patterns could also be related to the high %F of debris found in turtles in this area.

In the feces and gut contents analyses, green turtles ingested more artificial debris compared to loggerhead turtles. This trend was also reported in gut content analysis in previous studies^19^, however, the reason for the inter-specific difference in the amount of debris ingestion was not known. Our video data demonstrated that green turtles encountered more debris and that they were more attracted by the debris than loggerhead turtles. The encounter ratio difference between species is believed to be related to the vertical distribution of their diet. Video data indicate that the feeding depth of green turtles is shallower than that of loggerhead turtles. Most debris encountered by the turtles was drifting near the surface. Therefore, it is suggested that the green turtles encountered more debris because of their shallow feeding depths. Interestingly, the encounter-ingestion ratio also differed significantly between two species. It is thought to be causally related to shape and movement of their typical diet components. The loggerhead turtles mainly foraged on siphonophores, which are long thin organisms, and benthic animals, such as sea urchins and gastropods. On the other hand, the green turtles mainly foraged on marine algae and some scyphomedusa and salps, which are passively swaying and drifting materials. Moreover, their shapes are similar to transparent soft plastic debris. Hence, we suggested that loggerhead turtles could distinguish the artificial debris from actual food items when they approached due to the shape and movement of these items, whereas passively drifting debris might be hard to distinguish from common dietary elements for green turtles.

Artificial debris ingestion is considered as a significant stress for sea turtles^18^,^19^. However, it should be noted that ingestion of artificial debris does not necessarily have immediate lethal effect, as it appears that sea turtles frequently ingested natural debris such as stones, bird feathers and leaves, and a lot of natural and artificial debris are able to pass and excrete them. Despite these results, there is still considerable potential sub-lethal effect such as reduced food intake^5^ and transfer of toxic compounds^6^,^7^,^8^. Therefore, further research is required to understand the threat of debris ingestion on the health of sea turtles. Recently in the European Community, International standardized protocol for artificial debris characterization has been established^49^. This protocol is geared toward gut content and feces analysis, therefore it is possible that this protocol could not reflect the amount of debris which turtles encountered under natural condition. In addition to feces and gut content analyses, animal-borne video data can provide complementary information regarding debris ingestion under natural condition. This study documented that the gelatinous preys were more important diet for loggerhead and green turtles migrating to the Sanriku Coast which could be related to the high %F of debris found in turtles in this area. Additionally, we also demonstrated that the risk of debris ingestion was different between the species due to their different feeding habits. Our novel methodology which becomes important data for understanding the debris ingestion is directly applicable to various species, regions and seasons, and it could connect to establishing an effective mitigation plan.

## Materials and Methods

This study was performed in accordance with the guidelines of the Animal Ethic Committee of the University of Tokyo, and the protocol of the study was approved by this committee (A12-13, P13-6, P14-3, P15-7). This study was conducted as a part of tag and release program in which loggerhead and green turtles caught by set net as by-catch in the Sanriku Coast, were turned over by fisherman to researchers.

### Collecting feces and gut samples

Feces and gut samples were collected during July and October between 2012 and 2015. We collected turtles from fishermen when they were incidentally captured in coastal set-nets between Ofunato and Miyako on the Sanriku Coast (38°55′–39°40′N, 141°40′–142°05′E). All turtles were brought to the International Coastal Research Center (ICRC), Atmosphere and Ocean Research Institute, The University of Tokyo (39°21′05′′N, 141°56′04′′E) in Otsuchi town, Iwate prefecture, Japan. Turtles captured alive were kept in separate concrete tanks (3.6 × 1.5 × 1.0 m) for 1 day to up to 3 months and we checked these tanks every day. When feces were present in the tanks, they were collected using a net. To avoid missing of any pieces of feces on the bottom of tank, the drainpipe was placed near the surface. In addition, the water in the tank discharged into the drainpipe through the filter (1 cm). Dead turtles, only 2% and 5% of captured loggerhead and green turtles, respectively^33^,^34^, were dissected, and any materials found inside of the whole digestive tracts were collected. In addition, we also collected gut contents of dead turtles which had been released in the study area and subsequently recaptured by fisheries nets or stranded on beaches within 2 months after release. Feces and gut samples were either preserved frozen or in 99% ethanol. Samples were classified as diet items, natural debris and artificial debris. Diet items were identified to the class level by visual examination. Artificial debris were classified by types (hard plastic, soft plastic, Styrofoam, fishing line/rope, rubber and others) and colors (transparent, white, black and colored). Wet mass of each sample was weighed to 0.1 g using a digital scale. Then we determined percentages of frequency of occurrence (%F) and wet mass (%mass) for each type of item as follows:


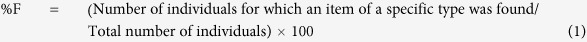



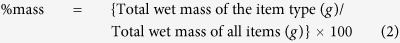


### Analysis of Bio-Logging data

To record foraging behavior of turtles, including the response behavior when they encountered artificial debris, animal-borne devices were attached to the carapace of 15 loggerhead and 10 green turtles in September and October during 2007 and 2015. Two types of video cameras were used in this study. One was the National Geographic “Crittercam” (76 mm in diameter, 350 mm in length, 1.5 kg in air for Gen. 5.5; and 57 mm in diameter, 230 mm in length, 0.8 kg in air for Gen. 5.7; National-Geographic Remote Imaging, Washington DC, USA^24^,^50^,^51^) which was used between 2007 and 2009 (the data set of loggerhead turtles were coincided with the data of Narazaki *et al*.^32^). The other was DVL400L (23 mm in diameter, 145 mm in length, 115 g in air; Little Leonardo Co, Tokyo, Japan) which was used between 2013 and 2015. In addition to these animal-borne video cameras, a 3D logger (W1000-3MPD3GT; 26 mm in diameter, 166 mm in length, 132 g in air, Little Leonardo Co, Tokyo, Japan) was used in all deployments to record tri-axial magnetism (1 Hz) and acceleration (16 or 32 Hz), swimming speed (1 Hz), depth (1 Hz) and temperature (1 Hz). The turtles were released around the Otsuchi Bay (39°20′N, 141°56′E) from 4–63 days after first by-catch. Data loggers were retrieved using auto-releasing system^52^,^53^,^54^.

Time-series data obtained from 3D loggers were analyzed using IGOR Pro ver 6.22 (WaveMatrics, Lake Osawago, OR, USA). 3D paths were calculated using data on depth, swim speed, acceleration, and magnetism obtained from the 3D loggers, as described by previous studies^53^,^54^,^55^. The 3D paths were reconstructed every 1 sec using a dead-reckoning method^53^,^56^,^57^,^58^. Video data were checked by using VLC media player (VideoLAN project: https://www.videolan.org) to identify any feeding events. The target of each feeding event was identified and the number of events for each type of item was counted. After linking 3D logger data, the depth of every event was noted. We also tallied both encounter and ingestion for artificial debris, and they were classified into types, colors and buoyancy (drifting or sinking).

### Statistics

Fisher’s exact test was used to examine whether the debris encounter-ingestion ratios of loggerhead and green turtles differed significantly. We examined the relationship between the artificial debris encounter rate and turtle species using generalized linear model (GLM). The dependent variable was the artificial debris encounter rate (encounters per hour), and the explanatory variable was the species (loggerhead or green turtles). We used the log link function. The most parsimonious model was selected on the basis of Akaike Information Criterion (AIC). All statistical analyses were performed using R^59^. Mean ± s.d. are presented unless otherwise indicated. The significance level of all statistical tests was set at α < 0.05.

## Additional Information

**How to cite this article**: Fukuoka, T. *et al*. The feeding habit of sea turtles influences their reaction to artificial marine debris. *Sci. Rep*. **6**, 28015; doi: 10.1038/srep28015 (2016).

## Supplementary Material

Supplementary Information

Supplementary Movie S1

Supplementary Movie S2

Supplementary Movie S3

Supplementary Movie S4

Supplementary Movie S5

## Figures and Tables

**Table 1:** Percentage of frequency of occurrence (%F) and wet mass (%mass) of different taxa and other materials in feces and gut samples obtained at the Sanriku Coast.

Category	Loggerhead turtles				Green turtles			
	%F		%mass		%F		%mass	
	Feces (n = 28)	Gut (n = 13)	Feces (n = 28)	Gut (n = 13)	Feces (n = 25)	Gut (n = 10)	Feces (n = 25)	Gut (n = 10)
Diet items								
Animalia								
Cnidaria								
Scyphozoa	0.0	15.4	0.0	7.4	0.0	0.0	0.0	0.0
Mollusca								
Bivalvia	32.1	23.1	10.7	8.3	0.0	0.0	0.0	0.0
Gastropoda	32.1	53.9	19.1	4.5	4.0	10.0	0.0	0.0
Cephalopoda	7.1	0.0	4.9	0.0	0.0	0.0	0.0	0.0
Arthropoda								
Malacostraca	35.7	38.5	0.9	8.7	4.0	10.0	0.0	0.0
Maxillopoda	28.6	61.5	23.2	16.5	72.0	90.0	9.6	2.9
Echinodermata								
Echinoidea	25.0	46.2	23.0	33.1	0.0	0.0	0.0	0.0
Chordata								
Osteichthyes	25.0	0.0	0.9	0.0	0.0	0.0	0.0	0.0
Ascidiacea	3.6	0.0	2.6	0.0	0.0	0.0	0.0	0.0
Others	25.0	69.2	0.5	4.0	4.0	60.0	0.0	0.9
Plantae								
Phycophyta								
Florideophyceae	7.1	0.0	0.2	0.0	36.0	100	3.8	20.2
Phaeophyceae	35.7	38.5	6.9	8.2	60.0	100	23.2	33.6
Ulvophyceae	10.7	15.4	0.9	0.3	12.0	80.0	3.0	12.1
Unknown	0.0	23.1	0.0	1.3	24.0	90.0	1.1	7.8
Angiospermae								
Monocotyledoneae	21.4	46.2	0.5	0.3	80.0	100	14.6	6.5
Natural debris								
Bird feathers	25.0	46.2	0.5	0.9	64.0	60.0	4.1	0.5
Wood/Leaves	28.6	69.2	1.9	1.0	12.0	50.0	0.4	1.1
Stones	28.6	30.8	1.4	0.4	4.0	50.0	0.1	0.3
Others	35.7	46.2	0.4	1.7	36.0	40.0	1.9	0.4
Artificial debris	35.7	84.6	1.5	3.6	100	100	38.6	14.3

**Table 2:** Percentage of frequency of occurrence (%F) and wet mass (%mass) of different types and colors of artificial debris in gut and feces samples obtained at the Sanriku Coast.

Category	Loggerhead turtles				Green turtles			
	%F		%mass		%F		%mass	
	Feces (n = 28)	Gut (n = 13)	Feces (n = 28)	Gut (n = 13)	Feces (n = 25)	Gut (n = 10)	Feces (n = 25)	Gut (n = 10)
Type								
Hard plastic	3.6	15.4	2.0	3.1	16.0	30.0	0.3	0.6
Soft plastic	28.6	53.9	67.6	52.7	92.0	100	76.7	88.9
Styrofoam	0.0	30.8	0.0	44.1	20.0	30.0	1.3	0.6
Fishing line/Rope	7.1	7.7	19.1	0.1	52.0	80.0	19.9	5.9
Rubber	0.0	0.0	0.0	0.0	4.0	40.0	0.1	0.4
Others	3.6	0.0	11.3	0.0	20.0	40.0	1.7	3.7
Color								
Transparent	28.6	53.9	61.5	51.0	80.0	100	46.2	55.6
White	7.1	30.8	20.4	45.8	64.0	100	29.3	18.6
Black	3.6	0.0	11.3	0.0	64.0	70.0	10.3	6.5
Colored	10.7	23.1	6.7	3.2	72.0	90.0	14.2	19.4

**Table 3:** Summary of deployments on loggerhead and green turtles at the Sanriku Coast, Iwate, Japan, between 2007 and 2015.

Turtle ID	Year	SCL (cm)	BM (kg)	Sex	Logger type**	Durationof video data (hours)	N of feeding events on food items	N of ingested natural debris	N of encountered artificial debris	N of ingested artificial debris
Loggerhead turtle										
L0704	2007	70.0	60.5	U	C1 + 3D	4.5	1	0	0	0
L0705	2007	80.0	83.0	U	C1 + 3D	3	1	0	1	0
L0708	2007	73.0	54.5	U	C1 + 3D	4.5	20	0	1	0
L0711*	2007	85.0	94.5	U	C1 + 3D	1	0	0	1	0
L0711*	2007	85.0	94.5	U	C1 + 3D	5	0	0	0	0
L0801	2008	77.8	63.0	U	C2 + 3D	3	2	0	0	0
L0947	2009	78.1	77.0	M	C2 + 3D	3.5	47	0	2	0
L1401	2014	72.4	54.0	U	DVL + 3D	12	0	0	1	0
L1410	2014	80.4	65.0	M	DVL + 3D	11.5	4	0	4	2
L1411	2014	80.7	65.0	M	DVL + 3D	12	6	1	2	0
**Total**						**60**	**81**	**1**	**12**	**2**
Green turtle										
G0718	2007	81.0	70.5	U	C1 + 3D	3	12	0	0	0
G0812	2008	60.4	31.0	U	C2 + 3D	3.5	21	0	1	0
G1354	2013	44.5	10.5	U	DVL + 3D	12	19	7	1	1
G1356	2013	48.4	14.0	U	DVL + 3D	11.5	104	1	0	0
G1454	2014	47.3	16.0	U	DVL + 3D	11	4	6	17	11
G1514	2015	49.3	19.0	U	DVL + 3D	11.5	4	11	15	9
**Total**						**52.5**	**164**	**25**	**34**	**21**

^*^L0711 was used for the study twice because it was recaptured by a set net after the first deployment.

^**^Abbreviations were used for logger type: C1 (Crittercam Gen. 5.5), C2 (Crittercam Gen. 5.7), DVL (DLV400L) and 3D (W1000-3MPD3GT).

**Table 4:** Percentage of frequency of occurrence (%F) and number of feeding events of different taxa and other materials in animal-borne video camera for the turtles released at the Sanriku Coast.

Category	Loggerhead turtles (n = 10)		Green turtles (n = 6)	
	%F	N of events	%F	N of events
Diet items				
Animalia				
Cnidaria				
Scyphozoa	50.0	11	50.0	6
Hydrozoa	40.0	67	0.0	0
Arthropoda				
Malacostraca	10.0	1	0.0	0
Maxillopoda	10.0	1	0.0	0
Chordata				
Thaliacea	0.0	0	16.7	2
Plantae				
Phycophyta				
Florideophyceae	0.0	0	33.3	73
Phaeophyceae	0.0	0	83.3	29
Ulvophyceae	0.0	0	33.3	8
Unknown	10.0	1	66.7	46
Natural debris				
Bird feathers	0.0	0	33.3	4
Wood/Leaves	10.0	1	66.7	21
Artificial debris	10.0	2	50.0	21

**Table 5:** Number of encounter/ingested debris of different types, colors and buoyancy.

Category	Loggerhead turtles (n = 10)		Green turtles (n = 6)	
	N of encountered	N of ingested	N of encountered	N of ingested
Type				
Hard plastic	1	0	0	0
Soft plastic	8	0	26	17
Styrofoam	1	1	1	0
Fishing line/Rope	2	1	5	3
Rubber	0	0	2	1
Color				
Transparent	5	0	23	15
White	4	1	4	2
Black	1	0	1	1
Colored	2	1	6	3
Buoyancy				
Sinking debris	2	0	0	0
Drifting debris	10	2	34	21
