# Supplementary material for: The feeding habit of sea turtles influences their reaction to artificial marine debris
Source: Sci Rep. 2016 Jun 16;6:28015. doi: 10.1038/srep28015 (PMC4910051; doi:10.1038/srep28015)
Supplement: Supplementary Information [file srep28015-s1.pdf]

Supplementary Information

The feeding habit of sea turtles influences their reaction to artificial marine debris

***Takuya Fukuoka[1], Misaki Yamane[1], Chihiro Kinoshita[1], Tomoko Narazaki[1], Greg J. Marshall[2], Kyler J. Abernathy[2], Nobuyuki Miyazaki[3], Katsufumi Sato[1]**

Table S1 The result of generalized linear model investigating the relationship between encounter rate and turtle species.

GLM model		AIC	Δ AIC
Encounter rate	~ 1	128.8	39.63
	~ Species	89.17	-

Movie S1 A loggerhead turtle (L1410) foraged a blue crab.

(mov)

Movie S2 A loggerhead turtle (L1410) consumed gooseneck barnacle on the Styrofoam

buoy.

(mov)

Movie S3 A green turtle (G1454) fed on a jellyfish.

(mov)

Movie S4 A green turtle (G1514) fed on a salp.

(mov)

Movie S5 A green turtle (G1454) ingested the artificial debris.

(mov)
